# Osmotic gradients and transretinal water flow—a quantitative elemental microanalytical study of frozen hydrated chick eyes

**DOI:** 10.3389/fncel.2022.975313

**Published:** 2022-10-24

**Authors:** Alan T. Marshall, Sheila G. Crewther

**Affiliations:** ^1^Analytical Electron Microscopy Laboratory, Department of Genetics and Environment, La Trobe University, Melbourne, VIC, Australia; ^2^Department of Psychology and Counselling, La Trobe University, Melbourne, VIC, Australia

**Keywords:** elemental microanalysis, transretinal fluid flow, retinal osmoregulation, intracellular osmotic concentrations, ion and water gradients, ion transporters, apical and basal RPE membrane gradients, taurine contribution to osmotic gradients

## Abstract

Optical clarity and efficient phototransduction are necessary for optimal vision, however, how the associated processes of osmoregulation and continuous fluid drainage across the whole eye are achieved remains relatively unexplored. Hence, we have employed elemental microanalysis of planed surfaces of light-adapted bulk frozen-hydrated chick eyes to determine the unique intracellular elemental localization, compositions, and hydration states that contribute to maintaining osmotic gradients and water flow from the vitreous, across the retina, retinal pigment epithelium (RPE), to choroid and sclera. As expected, the greatest difference in resultant osmotic concentration gradients, [calculated using the combined concentrations of sodium (Na) and potassium (K)] and tissue hydration [oxygen-defined water concentration], occurs in the outer retina and, in particular, in the RPE where the apical and basal membranes are characterized by numerous bioenergetically active, osmoregulating ion transport mechanisms, aquaporins, and chloride (Cl) channels. Our results also demonstrate that the high intracellular Na^+^ and K^+^ concentrations in the apical region of the RPE are partially derived from the melanosomes. The inclusion of the ubiquitous osmolyte taurine to the calculation of the osmotic gradients suggests a more gradual increase in the osmotic transport of water from the vitreous into the ganglion cell layer across the inner retina to the outer segments of the photoreceptor/apical RPE region where the water gradient increases rapidly towards the basal membrane. Thus transretinal water is likely to cross the apical membrane from the retina into the RPE cells down the Na^+^ and K^+^ derived osmotic concentration gradient and leave the RPE for the choroid across the basal membrane down the Cl^−^ derived osmotic concentration gradient that is sustained by the well-described bioenergetically active RPE ion transporters and channels.

## Introduction

The eye is arguably the most important sensory organ guiding higher vertebrate behavior. Yet despite the vast literature relating to the reception of light, phototransduction, and processing of visual information in the eye, relatively little is known about how optical clarity of the globe and retina (Marmor, 1997; Stone and Flitcroft, 2004) is maintained or how homeostatic size of the eye is achieved. Indeed, the retina, in particular, is known to have the greatest metabolic demand of any tissue in the body as it supports the major molecular and physiological changes in neurotransmission that accompany both the “dark current” (Dmitriev et al., 1999) at night and continuous light/dark transitions during the day (Country, 2017). The eye, as a closed system, requires strict homeostatic control of access to oxygen and glucose and removal of metabolites including large amounts of water associated with cellular function. In addition to the need for continuous drainage of water associated with the neural elements of the retina and neurotransmission (Wimmers et al., 2007; Country, 2017) there is an even greater need for the removal of the >20% of the aqueous fluid secreted by the ciliary body that flows around the lens, vitreous gel, and transretinally towards the retinal pigment epithelium (RPE) and vascular choroid (Marmor, 1988, 1990, 1997; Strauss, 2005; Smith et al., 2020). The eye is also subject to circadian variation in intraocular pressure (Liu et al., 1998; Liu, 1998), that induces circadian variation in axial elongation (reviewed by Lauber and Shutze, 1964, and Chakraborty et al., 2018) and refractive status in chick (Lauber and Shutze, 1964; Weiss and Schaeffel, 1993) and other animals including humans (Stone and Flitcroft, 2004) suggesting that transretinal fluid flow is greater in normal modulating light periods than in the dark (Reichhart and Strauß, 2020). Importantly, persistent prolonged accumulation of fluid in the vitreal chamber of the eye is clinically associated with myopia in the young (Holden et al., 2016) and with a number of other severe secondary ophthalmic disorders such as age-related macular degeneration, macular edema, and retinal detachment later in life (Stern et al., 1980).

Exactly how adequate ocular fluid drainage is achieved and how individual biologically important elements in the posterior eye may contribute to transretinal osmotic flow is unknown (Hamann, 2002) despite extensive neuroanatomical (Cajal, 1892; De Robertis and Lasansky, 1965; Lasansky, 1965; Dowling, 1970; Dreher et al., 1994) and electrophysiological (Miller and Steinberg, 1977; Sieving and Steinberg, 1985; Steinberg, 1985; Wimmers et al., 2007) investigations in relation to the ionic control of neuronal processing and fluid movements during light-dark modulation (reviewed for many species in Gallemore et al., 1997, and Straub, 2014). Morphological techniques such as electron microscopy and elemental microanalysis in chicks (Liang et al., 1995, 2004; Junghans et al., 1999; Crewther et al., 2006) and Time of Flight Secondary Ion Mass Spectroscopy (ToFSIMS; Gong et al., 2002), Particle Induced X-ray Emission (PIXE) and synchrotron x-ray fluorescence in rodent retina (Sergeant et al., 2001; Ugarte et al., 2012, 2014; Grubman et al., 2016) have all demonstrated a layered distribution of the elements sodium (Na), potassium (K), chloride (Cl), and nitrogen (N) in fixed and freeze-dried retinal preparations. Trace elements calcium (Ca), zinc (Zn), iron (Fe), K, Ba, and copper (Cu) have also been localized in the pigment granules of the apical microvilli of the RPE and choroid (e.g., Panessa and Zadunaisky, 1981; Samuelson et al., 1993; Biesemeier et al., 2011a, b). However, how this layered distribution of elements in fixed or dried samples, contributes to osmotic gradients and water movements in cellularly hydrated states is largely unknown with only pilot data on element distribution in frozen-hydrated samples of the normal chick retinal complex currently available in a recent methodological study (Marshall and Crewther, 2021) and a short exploratory analysis of the choroidal vessels by Wadley et al. (2002).

Certainly, the dynamic role of the basement membranes and sodium-potassium ATPase (NaK-ATPase) RPE ion channels (Na, K, and Cl ions) in rapid light/dark transitions, has been extensively investigated physiologically and shown to create an osmotic trans-epithelial potential (TEP) gradient between the basal and apical membranes that leads to movement of ions, water, and metabolic products from the hyperosmotic subretinal space (SRS)/apical RPE regions of the outer retina via the chloride channels on the RPE basal membranes (see review Gallemore et al., 1997) towards the vascular choroid. Hamann (2002), also noted that osmosis alone could not account for the movement of water from the retinal compartment to the choroid because the retinal compartment is hyperosmotic to the choroid, possibly due to lactate in the RPE (Hamann, 2002).

Water transport in RPE has also been associated with relative concentrations of taurine as modulated by subretinal space K^+^ (Orr et al., 1976; Huxtable, 1992; Schaffer et al., 2000; El-Sherbeny et al., 2004; Hillenkamp et al., 2004a, b) epinephrine (Edelman and Miller, 1991), and GABAAR (Cesetti et al., 2011) that are all predominantly associated with Cl^−^ transport by basal Cl channels (Gallemore et al., 1997; Dmitriev et al., 1999). Chloride channels are particularly abundant in both membranes of the RPE (Gallemore et al., 1997; Dmitriev et al., 1999). Interestingly excessive eye size that is the hallmark of clinical myopia and form deprivation myopia in animal models has also been shown in chicks using RNA-seq genomics and Gene Set Enhancement Analysis, to be primarily associated with suppression of ligand-gated chloride efflux channels including GABA_A_, GABA_C_ and Glycine channels and taurine pathways (Vocale et al., 2021) that are reversed after form deprivation is ended and normal vision initiated.

Osmotic regulation of the posterior eye has also been associated with intracellular elements such as phosphorus (P) and sulfur (S)—major components of nucleic acids, phospholipids, and metabolites [e.g., adenosine triphosphate (ATP)] and other organic phosphates (Gilles, 1979), paracrine molecules such as taurine, epinephrine, and glutamate and blood-borne hormones (Gallemore et al., 1997). S is also the major constituent of taurine that is the most common, and important intracellular osmolyte (Schaffer et al., 2000; Netti et al., 2018) of the retina (Orr et al., 1976; Ripps and Shen, 2012) and brain (Huxtable, 1992; Nagelhus et al., 1994; Pasantes-Morales and Schousboe, 1997). Taurine is one of the four essential non-charged (Netti et al., 2017) S containing amino acids and is involved in cellular processes such as energy metabolism, gene expression, osmosis, and quality control of protein and is known to be essential for the maintenance of retinal integrity and especially that of the outer retina in cat (Ripps and Shen, 2012). Taurine together with glutamate (Netti et al., 2018), contributes to cell volume regulation in human retinal Müller cells (Guizouarn et al., 2000; Netti et al., 2017, 2018). Earlier work has shown taurine to be concentrated in the retinal outer nuclear layer by a Na-dependent mechanism and released into the subretinal space following light onset (El-Sherbeny et al., 2004; Hillenkamp et al., 2004a, b). Light onset depolarizes the apical membrane, and activates the Na/K pump while actively co-transporting taurine with Na into the RPE (El-Sherbeny et al., 2004; Hillenkamp et al., 2004a, b). Hillenkamp and colleagues have also established that the magnitude and direction of taurine transport from the choroid into RPE and then the retina is modulated by subretinal space levels of K (Hillenkamp et al., 2004a, b) and that taurine transporter (TauT) levels are regulated by hyperosmolarity (El-Sherbeny et al., 2004) and contribute to fluid efflux transretinally.

Thus, the primary aim of the present investigation was to determine quantitatively, by chemical imaging of frozen-hydrated and freeze-substituted samples: (i) the structural evidence of static intracellular elemental, and hydration composition of the various layers of the retina; and (ii) how the relative concentration of the ions across the retina contributes to the osmoregulatory gradients that maintain optical clarity and fluid efflux from the vitreous to the RPE and choroidal vasculature.

Our secondary aim was: (iii) to enhance understanding of relative tissue hydration and the Na and K, osmoregulatory gradients that contribute to retinal fluid movements associated with Cl ions from vitreous to the choroid. Lastly, we aimed (iv) to model the influence of taurine content, as measured in chick by Orr et al. (1976), on the transretinal osmotic gradients needed to maintain optical clarity, retinal integrity, and neural transmission of the eye.

## Methods

Five male chicks *Gallus gallus domesticus* (Leghorn/New Hampshire) were raised under a 12-h day/night light cycle from post-hatch day 1 until anesthetized 5 h into the light cycle on day 5. Surgical anesthesia was induced by intramuscular injection of a mixture of ketamine (45 mg kg^−1^) and xylazine (4.5 mg kg^−1^) and right eyes were enucleated prior to death by anesthetic overdose. After enucleation, the vitreous humor of the eye was removed and the posterior eye cut into slices (about 2 × 4 mm) of retina-sclera tissue complex that was rapidly frozen by plunging into liquid propane cooled by liquid nitrogen to around 87°K. Three left eyes were frozen intact by plunging into liquid propane. Samples were then stored in liquid nitrogen until required.

All procedures were conducted in accordance with the protocols approved by the La Trobe University Animal Ethics Committee and adhered to the ARVO Statement for the use of animals in ophthalmic and vision research.

### Frozen-hydrated samples

Analyses of frozen-hydrated samples were carried out in a JEOL JSM840A SEM (JEOL Australasia Pty Ltd, Frenchs Forest, NSW, Australia) as in Marshall (2017) and Marshall and Crewther (2021). The preparation success rate was approximately 60%. Briefly, qualitative elemental images (maps) presented as x-ray counts, corrected for background and spectral overlaps, and quantitative images presented as weight percent were obtained at 15 kV with a beam current of 2 × 10^−10^ A over a period of 18–20 h at a resolution of approximately 1–3 pixels per micron. It should be noted that x-ray microanalysis measures total element concentrations and cannot distinguish between bound and ionized elements. Thus, elemental symbols in the ionized form are only used when referring to physiological processes that do depend on the actual ionic concentrations as in the Nernstian sense whereas EDS can only actually look at elemental abundance—ionic or bound.

Quantitation (Marshall, 2017) was carried out on spectra extracted from selected regions on elemental maps, as described in Marshall and Crewther (2021). The latter authors showed that selected areas of the retina can give identical results to the analysis of individual cells and that at an accelerating voltage of 15 kV, O x-rays are largely derived from intracellular water. Concentrations are given in weight percent i.e., mass fraction (mass of element per analyzed mass; Marshall, 1975; Heinrich, 1991) by the software and converted to mmol kg^−1^ wet weight as required. The conversion from mmol kg^−1^ wet weight of tissue to mmol l^−1^ of intracellular water requires H_2_O concentration to be derived from O concentration. The latter was accomplished by applying Equation (1; Marshall et al., 2012).


(1) $${\text{H}}_{\text{2}}\text{O = −33.242 + 1.49706 * O concentration}$$

Equation (1) will be less accurate when applied to the cartilaginous sclera where cells are surrounded by an extensive matrix of glycosaminoglycans.

Intracellular osmotic concentrations were calculated by Equation (2) after Schmidt-Nielsen (1976)


(2) $${\text{mosmol l}}^{-1}=({\text{Na + K) mmol l}}^{-1}*1.85$$

The osmotic concentration of the glycosaminoglycan matrix of the sclera is difficult to calculate (Chahine et al., 2005). An estimate of osmotic concentration was calculated by using osmotic coefficients for NaCl and Na_2_SO_4_ (Equation 3).


(3) $${\text{mosmol l}}^{-1}=({\text{Na) mmol l}}^{-1}*1.67$$

Quantitative line-scans in weight percent were extracted from elemental maps.

Quantitative data are graphically displayed as means and standard deviations (SD). The small sample size did not justify further statistical comparisons (Vaux, 2012; Button et al., 2013; Nuzzo, 2014; Halsey et al., 2015).

### Freeze-substituted samples

Frozen samples were also freeze-substituted (FS) using a method designed to retain diffusible elements. Briefly, samples were freeze substituted in 10% acrolein in diethylether, essentially as described by Marshall (1980), infiltrated in increasing concentrations of ether and Araldite^TM^ mixtures and embedded in Araldite^TM^. Araldite^TM^ was the preferred embedding medium as it contains negligible levels of elements detectable by energy dispersive spectroscopy (Palsgard et al., 1994a, b). All solutions were anhydrous, with processing conducted in a dry box at a relative humidity of 10%. Dry cut sections 1.0–2.0 μm thick were mounted on a nylon film on custom-made supports for analysis in the JEOL 840A SEM operated in scanning transmission electron microscopy (STEM) mode at 40 kV and a beam current of 2 × 10^−10^ A.

Qualitative elemental images were made from freeze-substituted sections of the isolated retinal complex using the SEM operating in STEM mode to take advantage of both the increased optical resolution and the signal peak to background ratio that results in improved detection of higher atomic number trace elements. This is due to the higher accelerating voltage of the STEM permitting the use of the higher critical ionization energy of the K line peaks in the x-ray spectrum, rather than the low energy lines that have to be digitally separated from overlapping K line peaks of low atomic number elements.

## Results

### Elemental distribution across frozen-hydrated samples of normal eyes

**Figure 1:**
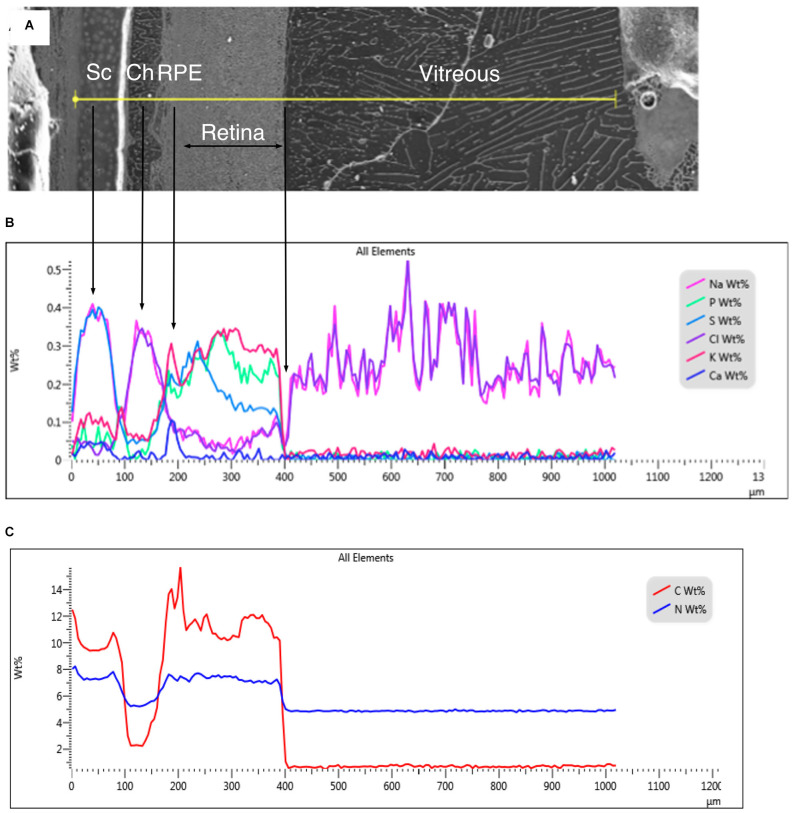
**(A)** Secondary electron image of a region of frozen-hydrated chick eye. Sc, sclera; Ch, choroid; RPE, retinal pigment epithelium. The yellow line indicates the position of the line-scan in **(B)**. **(B)** Quantitative line-scan of various elements in weight percent. **(C)** Quantitative line-scans of N and C in weight percent.

**Figure 2:**
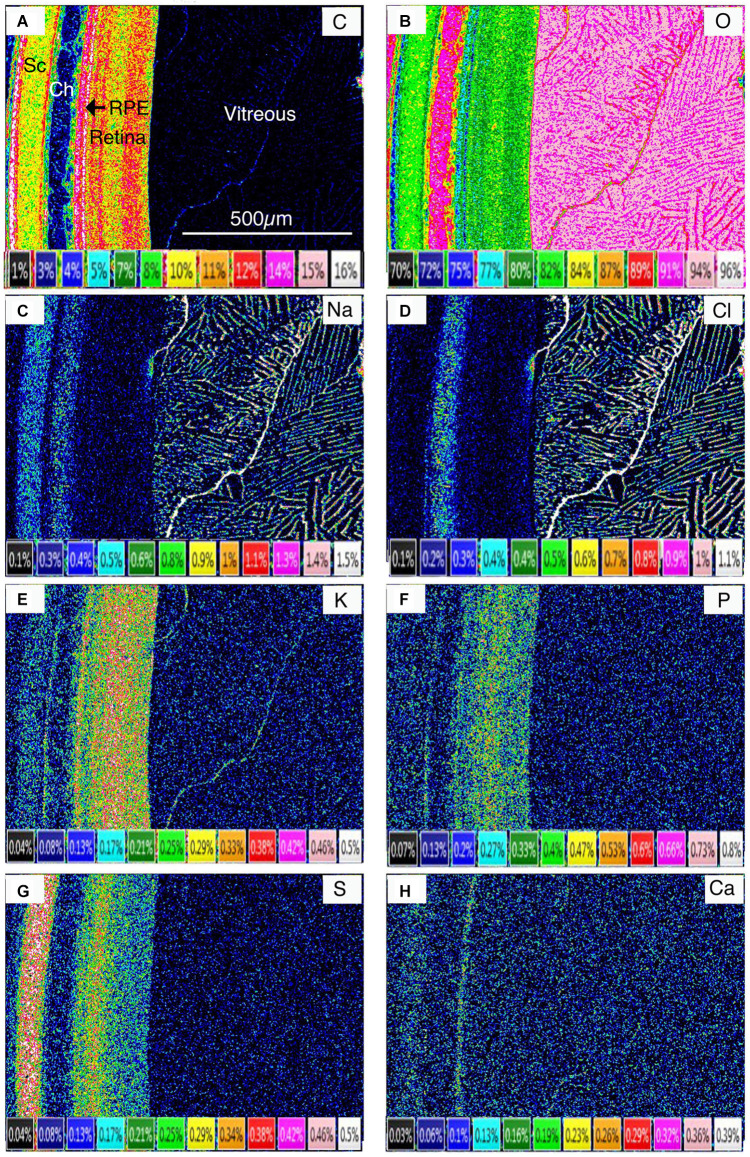
Quantitative elemental concentration images in weight percent of frozen-hydrated chick eye. Sc, sclera; Ch, choroid; RPE, retinal pigment epithelium. **(A)** Carbon (C). **(B)** Oxygen (O). **(C)** Sodium (Na). **(D)** Chlorine (Cl). **(E)** Potassium (K). **(F)** Phosphorus (P). **(G)** Sulfur (S). **(H)** Calcium (Ca).

Intact frozen eyes were fractured into smaller pieces for cryoplaning. The size of intact eyes precluded sufficiently rapid cooling to prevent the formation of large size ice crystals, particularly in the vitreous humor (Figure 1A). Furthermore, it is impossible to prevent some variation in the x-ray signal due to the widely separated solute segregation zones. Quantitative elemental images (Figure 2) of a region including that in Figure 1A show the distribution of elements across the entire posterior eye including the vitreous chamber and retina-RPE-choroid sclera complex. Even at this low magnification, the layered diversity of elemental concentrations across the retinal complex is evident and is well displayed in quantitative line-scans (Figure 1B). Variation in the Na and Cl concentrations across the vitreous fluid is thought to be due to variation in ice crystal size and density of the segregation zones. The determination of absolute concentration values was not possible due to the difficulty of controlling the ice sublimation rate of a large sample mass. Nevertheless, it can be seen that Na and Cl concentrations in the vitreous gel were similar in the fluids in the choroid vessels. As shown in Figure 1C, the concentration of nitrogen (N) in the vitreous fluid was markedly higher than the concentration of C.

Given that the distribution of Carbon (C) is a proxy for tissue mass, and that of Oxygen (O) a proxy for H_2_O concentration, Figure 2 clearly shows that the hydration state across the retinal complex, like the elements, varies between layers (Figure 2).

**Figure 3:**
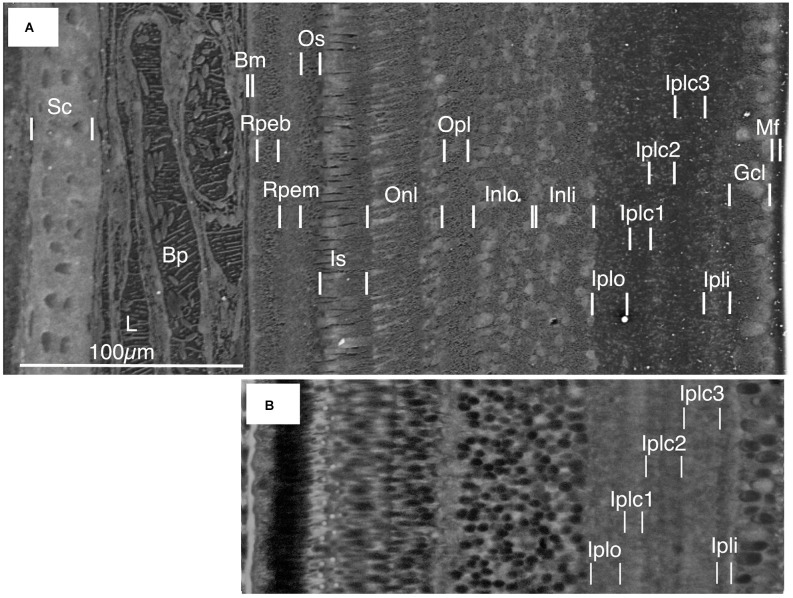
**(A)** Frozen-hydrated retinal complex showing cell and tissue layers. Sc, sclera; L, frozen lymph within the lymphatic vessel; Bp, frozen blood plasma within blood vessel containing erythrocytes; Bm, Bruch’s membrane, Rpeb, basal region of the RPE (retinal pigment epithelium); Rpem, apical melanosome-containing region of RPE; Os, outer segment region of photo receptors; Is, inner segment of photoreceptors; Onl, outer nuclear layer; Opl, outer plexiform layer; Inlo, inner nuclear layer outer region; Inli, inner nuclear layer inner region; Iplo, inner plexiform layer outer layer; Iplc1, inner plexiform layer central layer 1; Iplc2, inner plexiform layer central 2; Iplc3, inner plexiform layer central 3; Ipli, inner plexiform layer inner layer; Gcl, ganglion cell layer; Mf, Muller cell feet and nerve layer. **(B)** Stained semithin section of freeze-substituted retina and RPE. The cell layers correspond to those in the frozen-hydrated retinal complex in **(A)**.

**Figure 4:**
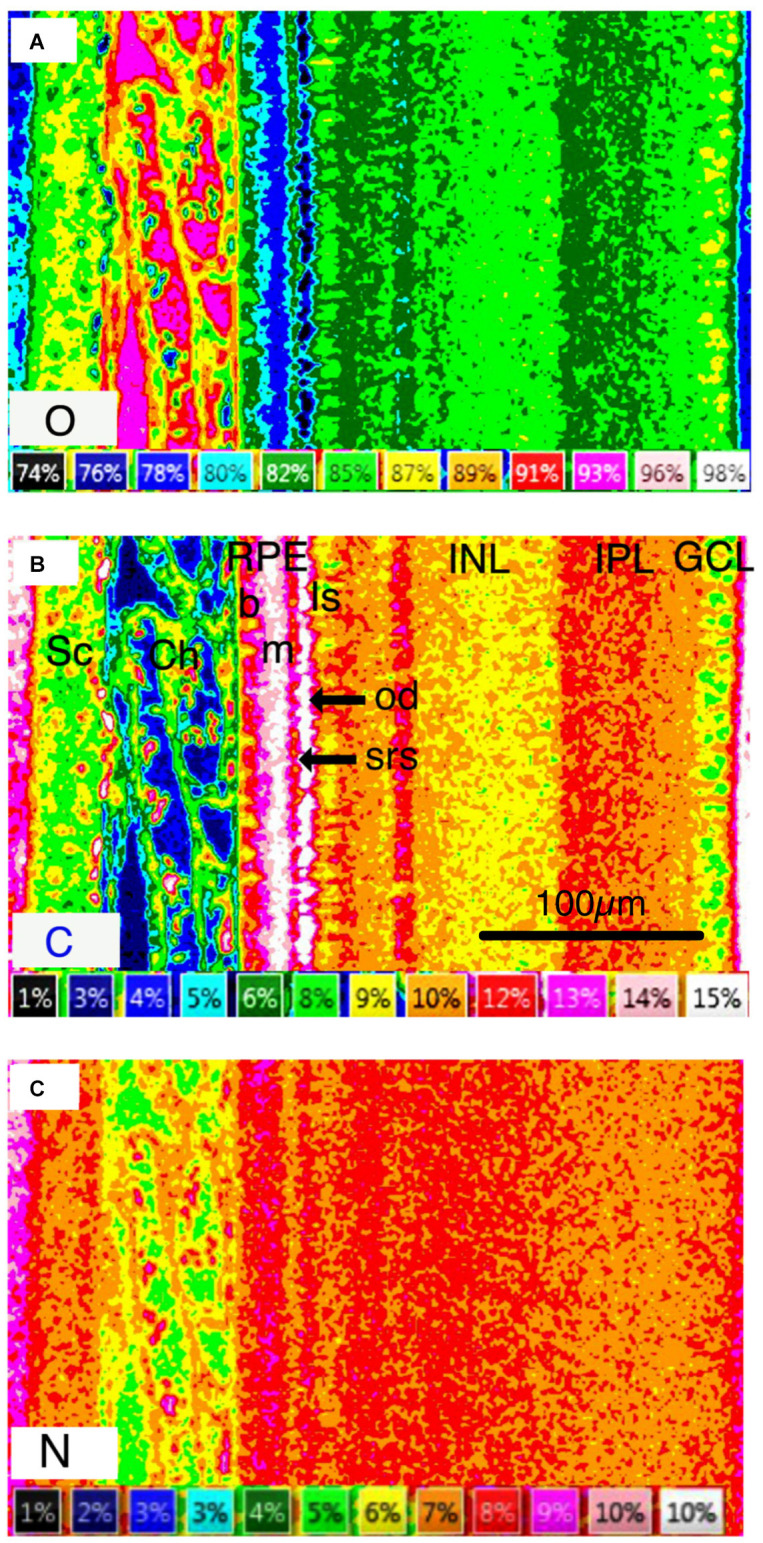
Quantitative concentration images of frozen-hydrated retinal complex in weight percent. **(A)** Oxygen [O] concentration is a proxy for water concentration. **(B)** Carbon [C] concentration image of frozen-hydrated retinal complex in weight percent. Carbon concentration is a proxy for organic mass. **(C)** Nitrogen [N]. Abbreviations: O, oxygen; C, Carbon; Sc, sclera; Ch, choroid; RPE, retinal pigment epithelium; b, basal region of RPE; m, apical melanosome-containing region of RPE; Is, inner segment of photoreceptor layer; od, oil droplets; SRS, subretinal space; INL, inner nuclear layer; IPL, inner plexiform layer; GCL, ganglion cell layer. **(C)** Nitrogen [N] concentration image.

Improved preservation and higher resolution images were obtained from cryo-planed frozen pieces of isolated retinal complex (Figures 3A and 4A). The following structural elements were discernible: cartilaginous sclera (Sc) containing chondrocytes; choroid (Ch) containing lymph vessels (L) and blood vessels in which blood plasma (Bp) was distinguishable from red blood cells; Bruch’s membrane (Bm); retinal pigment cells (RPE) with the apical melanin-containing layer (RPEm) and basal nuclear layer (RPEb): a region of the outer segments (Os) of the photoreceptor cells (PR); inner segments (Is) of the photoreceptor cells together with the position of the outer limiting membrane (Olm) and the outernuclear layer (Onl); outer plexiform layer (Opl); inner nuclear layer (INL); inner plexiform layer (IPL) comprising five sub-layers; the ganglion cell layer (GCL) and the nerve fiber/Müller cell feet layer (Mf). A light micrograph of a stained semithin section of a similar sample of freeze-substituted retina shows the quality of preservation and the five IPL sublayers (Figure 3B).

**Figure 5:**
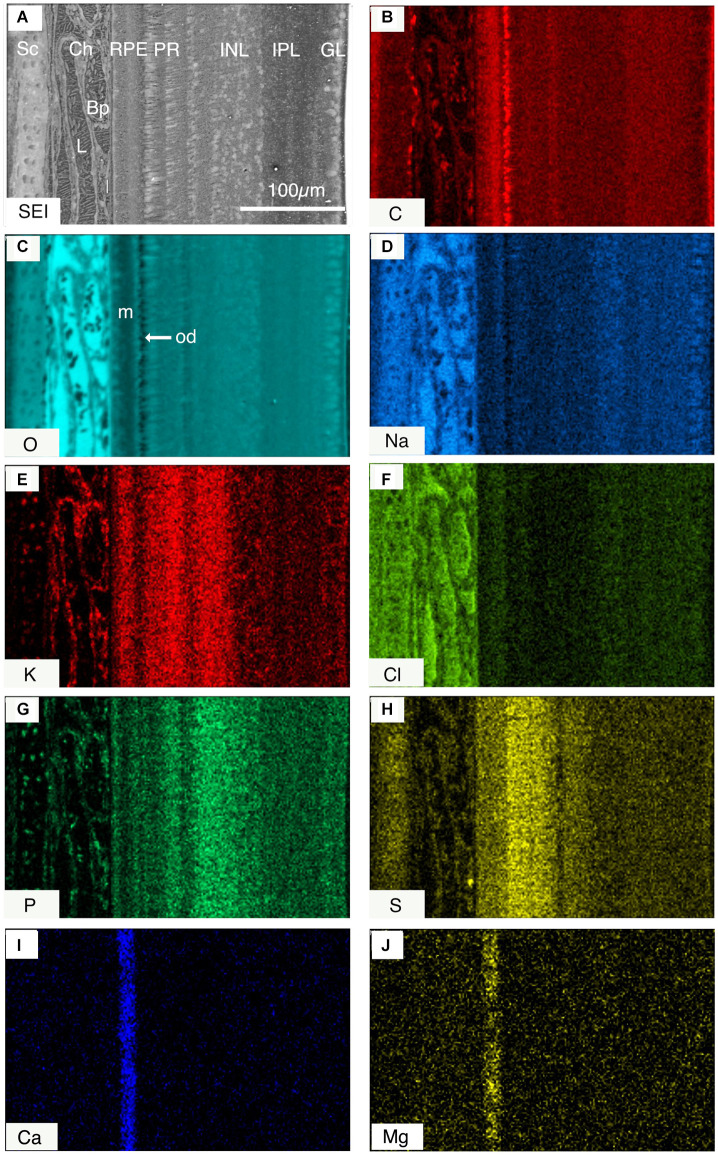
**(A)** Secondary electron image (SEI) across the frozen-hydrated retinal complex. **(B–H)** Qualitative images of the relative distribution of different elements **(B)** carbon C; **(C)** oxygen O, **(D)** sodium Na; **(E)** potassium K; **(F)** chlorine Cl; **(G)** phosphorous P; **(H)** sulfur S; **(I)** calcium Ca; and **(J)** magnesium Mg across the posterior eye, where each color represents x-ray intensity (counts) for a different element. The brighter parts of the images indicate higher concentrations of elements. Note **(I)** calcium, and **(J)** magnesium are predominantly localized in the melanosome-containing region of the RPE. Abbreviations: Sc, sclera; Ch, choroid; RPE, retinal pigment epithelium; PR, photoreceptor layer; INL, innernuclear layer; IPL, inner plexiform layer; GCL, ganglion cell layer; m, melanosome-containing region of RPE; od, oil droplet.

The layered nature of the element distribution is clearly apparent in quantitative elemental images of the major elements C, O, and N where concentration is expressed as weight percent (Figure 4), and in qualitative elemental images, where the color intensity is a measure of x-ray intensity and directly related to element concentration (Figure 5).

**Figure 6:**
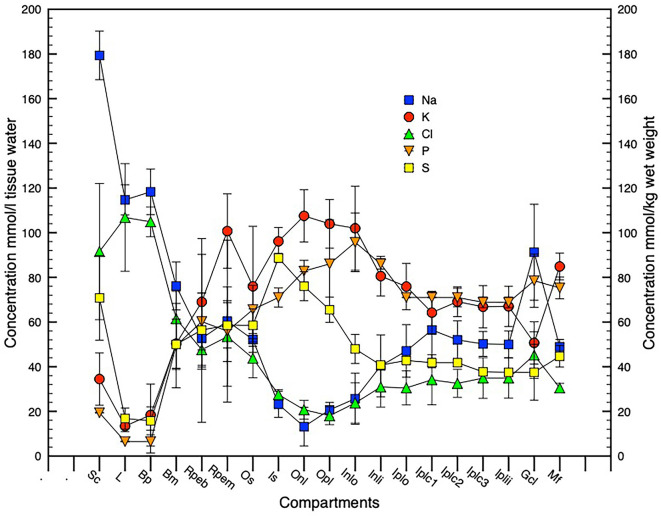
Graphical presentation of element concentrations across the retinal complex. N = 3, SD error bars. Na, K, Cl expressed in mmol l^−1^cell or tissue water and P, S expressed in mmol kg^−1^ wet cell or tissue mass. Compartment abbreviations on X-axis as in Figure 3A.

We have also quantified the elemental concentrations following extraction of the X-ray spectra from specific regions of elemental images (maps) of three samples, where the identification of cellular regions was unambiguous. These data together with data from individual ganglion cell analyses have been used to calculate concentrations of diffusible elements (Na, K, Cl) in all cell layers in mmol l^−1^ cell water. The other elements (P, S, Mg, Ca) were assumed to be largely bound and are represented as mmol kg^−1^ wet weight (Figure 6; Table 1). Not surprisingly Na and Cl concentrations are usually similar and closely aligned to percent weight of water in most layers. P as PO_4_ an essential anion is also usually present in similar concentrations to K (see Figure 6) concentrations except in the apical regions of the RPE (see Table 1). This positive correlation between intracellular potassium and phosphorus content has previously been noted in acanthamoeba (Sobota et al., 1984) and in rat liver and heart muscle (Von Zglinicki and Bimmler, 1987).

**Table 1:** Element concentration across the retinal complex with O concentrations converted to H_2_O concentrations.

		**Wt %**			**mmol/l**			**mmol/kg**			
		**C**	**N**	**H_2_O**	**Na**	**Cl**	**K**	**P**	**S**	**Mg**	**Ca**
Sclera	Mean	7	6.2	80.2	179.4	91.5	34.5	19.4	70.8	4.1	3.3
	SD	0.34	0.09	0.58	10.86	30.48	11.72	0.03	18.9	0.01	1.44
Blood Plasma	Mean	2.3	4.8	89.8	118.3	104.9	18.4	6.5	12.5	4.12	2.5
	SD	0.1	0.03	0.1	7.22	4.7	9.8	3.6	3.68	5.82	0
Lymph	Mean	1.5	2.7	91.2	114.7	106.8	13.4	6.5	14.3	2.1	1.3
	SD	0.06	2.83	0.21	6.76	24.1	2.94	0.04	2.47	2.91	1.77
Bruchs	Mean	7	6	80.8	75.2	61.5	50.3	50	49.8	4.1	0.8
	SD	1.7	0.79	3.25	10.74	12.24	11	15.24	11.25	4.11	1.44
RPE basal	Mean	8.9	6.3	77.4	52.7	47.6	69	65.6	56.4	13.7	9.2
	SD	2.27	0.56	4.3	37.59	31.88	28.41	9.85	16.63	6.3	7.64
RPE apical	Mean	11.7	6.8	72.3	60.4	53.5	100.7	55.9	58.5	24.7	37.5
	SD	1.13	0.05	1.79	36.24	22.25	16.63	9.84	10.15	8.22	4.33
RPE/OS	Mean	10.5	6.3	75	52.2	43.8	76	60.2	58.5	12.3	19.2
	SD	1.46	0.5	3.11	5.8	8.74	26.87	8.1	7.37	4.13	20.36
PR/Is	Mean	10.1	6.4	75.4	23.2	27.5	96.1	71	88.7	6.9	0.8
	SD	0.3	0.05	0.41	5.83	2.18	6.2	3.23	2.02	2.36	1.44
ONL	Mean	8.9	6.5	77	13.2	20.7	107.5	82.8	76	8.2	0.8
	SD	0.68	0.16	0.82	8.65	4.22	11.71	3.72	6.49	0.02	1.44
OPL	Mean	9	6.5	77	20.7	18.1	104	86	65.5	6.9	0.8
	SD	0.68	0.15	0.73	3.29	4.02	10.92	15.23	5.63	2.36	1.44
INL outer	Mean	7.7	6.47	78.9	25.7	23.8	102	95.7	47.9	6.9	0
	SD	0.27	0.19	0.81	11.45	8.97	18.8	10.36	6.5	2.36	0
INL inner	Mean	7.8	6.4	79.1	40.4	30.9	80.6	86	40.8	6.9	0
	SD	0.32	0.12	0.65	13.86	8.98	8.89	1.86	0.21	2.36	0
IPLOsl	Mean	9.2	6.3	77.2	47.1	30.5	75.9	71	42.8	4.11	0.83
	SD	0.12	0.12	0.38	11.76	7.63	10.34	0.02	1.88	0.01	1.44
IPLCsl1	Mean	9.2	6.2	77.3	56.5	34.1	64.2	71	41.8	4.11	1.67
	SD	0.14	0.11	0.33	16.93	11.18	9.51	0.02	1.7	0.01	1.44
IPLCsl2	Mean	9.1	6.1	77.6	52	32.5	69.1	71	41.8	5.5	2.5
	SD	0.18	0.1	0.39	8.5	6.27	6.7	3.23	1.7	2.38	0
IPLCsl3	Mean	9.1	6.14	77.6	52	32.5	69.1	71	41.8	5.4	2.5
	SD	0.18	0.1	0.39	8.5	6.27	6.7	3.23	1.7	2.4	0
IPLIsl	Mean	8.8	6.12	78.1	50.2	34.9	66.8	68.8	7.7	4.1	1.7
	SD	0.29	0.08	0.49	5.59	9.09	9.46	1.88	0.29	0.01	1.44
GCL	Mean	6.7	6.3	80.9	91.3	45.2	50.7	83.6	46.3	5.1	3.1
	SD	0.37	0.19	0.23	21.47	10.43	9.5	11.95	15.43	10.16	6.17
MFL	Mean	9.5	6.3	76.7	48.9	30.5	84.9	75.3	44.7	6.9	0
	SD	1.04	0.24	1.95	3.25	2.12	5.96	3.72	4.82	2.36	0

C, N, and H2O are expressed as Weight Percent, Na, K, Cl are expressed in mmol l^−1^ cell or tissue water and P, S, Mg, Ca are expressed in mmol kg^−1^wet cell or tissue mass*. N* = 3 SD standard deviation. Compartment abbreviations are as in Figure 3A. Numbers shown for the GCL analyses are for individual cells *N* = 9. RPE, retina pigmented epithelium basal and apical regions; RPE-OS, includes RPE microvilli that surround the outer segments (OS); and the subretinal space, IS, inner segment of photoreceptor; OS, outer segment of photoreceptor; INL, inner nuclear layer; IPL (OsL), Inner plexiform layer (outer sublayer); IPL (Csl, 2 and 3), Inner plexiform layer (central sublayers); and IPL (Isl), Inner Plexiform Layer (Inner sublayer); GCL, large ganglion cell; MFL, Muller cell end feet.

The cartilaginous sclera contained S, Na, and Cl in the matrix and P and K in the chondrocytes whilst the choroid contained, as expected, principally Na and Cl in lymph and blood vessels (Figures 5D,F). The elemental composition of the blood plasma and lymph were similar except for C (Figure 4B) and N (Figure 4C; plasma C 2.3, N 4.8 weight percent; lymph C 1.5, N 2.7 weight percent-Table 1) which suggests that plasma has a higher organic content than the lymph.

**Figure 7:**
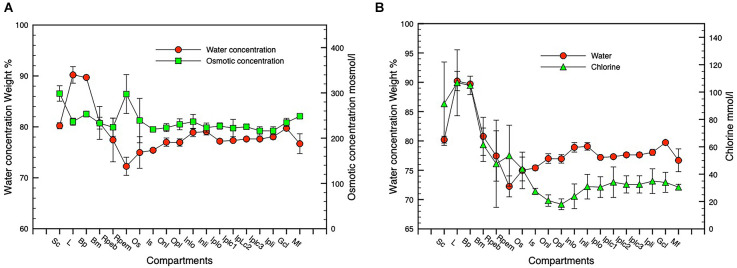
Water, osmotic, and chlorine concentration gradients. Panel **(A)** shows water and osmotic [(Na + K mmol l^−1^ * 1.85)] concentration gradients, panel **(B)** shows water and Cl concentration gradients. *n* = 3, SD error bars. Compartment abbreviations on X-axis as in Figure 3A.

The RPE at 5 h into the daylight cycle was characterized by a granular layer of high C concentration and low O concentration indicating a high organic mass and low water content (Figures 1, 4A,B, 5B,C, 6 and 7) in the melanosomes (m). Na and Cl were also strongly associated with the apical melanosome-containing region (Rpem) of the RPE cells and outer segments (OS) of the photoreceptor cells (PR; Figures 5D,F) whilst K and S were distributed across the RPE, including in the melanin layer (Figures 5E,H) with K somewhat higher in the apical region (Figure 6; Table 1). Calcium and Mg were also localized in the RPE melanin layer (Figures 5I,J; Table 1). Again, as alluded to earlier P is present in high concentrations when K is high and vice-versa in the RPE at the apical border.

The PR inner segments (IS) contained C-rich and O-poor granules (Figures 4A,B; 5B,C) identified as putative oil droplets. Outside of the oil droplets the IS showed a high O (H_2_O) content with intracellular concentrations of Na, K, and Cl, 23, 96, and 28 mmol l^−1^ respectively similar to the electrophysiological analyses of isolated frog RPE cells of K (110 mmo l^−1^) and Cl (20–60 mmol^−1^) of Miller and Steinberg (1977) and previous intracellular x-ray microanalysis ion concentrations of the OS and IS of isolated frog photoreceptors (Somlyo and Walz, 1985).

The results recorded above are compared and summarized with the technique indicated in Table 2.

Close inspection of Figures 5B,C; indicates that C-rich oil droplets are associated with moderately high intracellular O (H2O) in cone receptor inner segments. The photoreceptor IS and ONL contained high levels of K and S (Figures 5E,H) whilst the ONL also had a high) P content (Figure 5G). The outer plexiform layer (OPL) had a high K and P content (Figures 5E,G) whereas in the Na and Cl images only a faint, but distinct layer was evident (Figures 5D,F).

The innernuclear layers (INL) were characterized (Figures 4A,B,C; Figures 5B,C and Table 1) by high H_2_O (O) and a low C intracellular content of 79 and 7.8 percent, respectively, and high K and P, within the inner half (see 5C,E,G) of the nuclear-like structures of the INL (see Figure 5A). A positive association between the fluid excreting aquaporin4 (AQP4) receptors that lie predominantly on the proximal Müeller cells in the inner retina (Goodyear et al., 2008) is suggested. The distribution of S was lower in the vitreal facing half of the INL (Figure 5H) than in the outer retina, while the relatively lower content of K and S was mirrored by increases in the content of Na and Cl in this layer (Figures 5D,F). A possible interpretation is that the proximal, vitreal facing, half of the INL was occupied by the cell bodies of amacrine cells that have been associated with aquaporin AQP1 receptors in rat retina (Kim et al., 2002; Goodyear et al., 2008).

In the inner plexiform layer (IPL) P and S contents were uniformly low (Figures 5G,H) whilst Na tended to be high. Five sub-layers were identifiable in the secondary electron image of the IPL (Figures 3A and 4A) and in the light microscope image of freeze-substituted sections (3B). These layers varied slightly in elemental content. Carbon content was lower in the outer layers (Iplo) than in the inner sublayers whereas O (H_2_O) was higher in the outer layers than the inner (Figures 5B,C, 4A,B). The outer sub-layer (Iplo) had a higher K and lower Na and Cl content than the central sub-layer one (Iplc1) whilst Iplc2 had a higher K content than Iplc1. Both the inner sub-layer (Ipli) and Iplc3 had a lower K content than the Iplc2 and a similar Na and Cl content to Iplc1 and 2 but higher than the Iplo (Figures 5C–F).

The ganglion cell layer (GCL; Figure 4A) had a relatively high P, O (H2O) and high Na (91 mmol l^−1^) content and low C (7.5 weight percent) and 57 mmol l^−1^) contents (Figures 4A,B and 5B–E).

### Water and osmotic concentration across the posterior eye

As described in the methods the profile of water concentration across the retinal complex has been calculated from O concentrations that are presented in Table 1. Based on the assumption that the measured elemental concentrations in the retinal layers primarily reflect intracellular concentrations, a profile of osmotic concentration was calculated across the retinal complex and posterior eye (Figure 7A).

Figure 7A shows water and osmotic (Na + K) mmol l^−1^ * 1.85 *concentration gradients, Figure 7B shows water and Cl concentration gradients. n = 3, SD error bars. Compartment abbreviations on X-axis as in Figure 3A*.

Comparison of chlorine concentration and water concentration gradients across the posterior eye shows a relatively constant relationship across the inner retina that increases substantially in the outer retinal photoreceptor layer and in the RPE indicating a closer relationship between chloride channel activity and fluid flow across the RPE to the vascular choroid (Figure 7B).

*Freeze-substituted samples of normal eyes to permit improved imaging of high atomic number trace elements (see “Methods” section)*.

**Figure 8:**
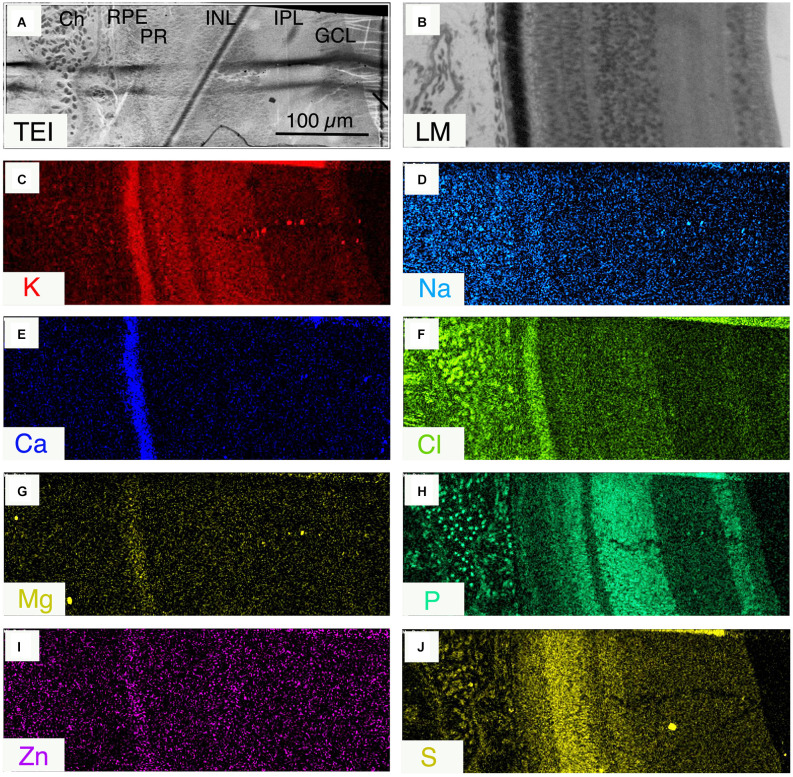
**(A)** Scanning transmission electron image (JEOL 840ASEM) of a 2 μm thick section of freeze-substituted retinal complex, Ch, choroid; RPE, retinal pigment epithelium; PR, photoreceptor layer; INL, inner nuclear layer; IPL, inner plexiform layer; GCL, ganglion cell layer. **(B)** Light micrograph of a stained section from the same sample as in **(A)**; **(C–J)** qualitative for ion indicated.

Detection of trace elements such as Zn was greatly improved in thick sections of freeze-substituted (FS) samples of isolated retinal complex in scanning transmission electron microscope (STEM) mode that enabled better optical resolution and signal peak to background ratio (see Figure 8A) than was possible using frozen hydrated tissue. Note the concurrent demonstration of Zn as well as Na, K, Cl, Ca, Mg, and S situated in the melanosomes in the microvillar apical region of the RPE (Figure 8I). Figure 8B shows a light micrograph of an FS section cut from the same STEM block evidencing the quality of the preservation. Figure 8 demonstrates that the elemental distribution patterns (Figures 8C–J), are essentially similar to frozen-hydrated (FH) samples Figures 3 and 5.

## Discussion

This manuscript is the first to report the distribution of water and elemental mass across the entire posterior complex of the normal chicken eye (sclera, choroid, RPE retina, and vitreous) in the light-adapted frozen-hydrated state as a basis for understanding osmoregulatory functions within the normal retina of vertebrate eyes. We have also demonstrated qualitative (Figures 5 and 8) and quantitative (Figures 2 and 4) elemental images and quantified the concentrations of the major elements of biological interest across the posterior eye, with concentration of C, N and O shown as weight percent, and Na, K, Cl as mmol l^−1^ cell water and P, S, Ca, and Mg as mmol.kg^−1^ wet weight. Furthermore, on the basis of the water gradient and combined concentrations of Na and K we have also calculated the hydration and osmotic concentration gradients across the entire posterior eye (see Figure 7) and shown that the greatest differences between the two gradients exist in the outer retina and particularly across the apical and basal regions of the RPE. This static anatomically derived finding concurs with dynamic physiological results regarding intracellular K^+^ driven osmotic gradients observed in many species (see Table 2) and reviewed by Gallemore et al. (1997), Crewther (2000), and Reichhart and Strauß (2020) implicating the need for bioenergetically maintained electrical gradients in the outer retina/RPE to achieve transretinal fluid efflux.

Elemental imaging and line-scanning of pieces of the intact frozen eye revealed 16 distinct retinal layers differing in elemental concentrations and composition (Figure 6) in addition to the RPE, inner vascular and outer lymphatic layers of the choroid and the collagenous layer of the sclera. In particular, the vitreous humor, although apparently heterogeneous in composition due to the differing densities of segregation of zones of ice crystals of different sizes, contained Na, K, and Cl at similar relative concentrations to the plasma contents of the choroidal blood and lymph vessels. Such vitreal concentrations of Na, K, and Cl are consistent with the ion selective electrode findings of Seko et al. (2000) though we also observed a much higher concentration of N than C in the vitreous than previously described, presumably due to the presence of significant quantities of nitrogen-containing compounds such as urea (Palmiere and Mangin, 2015).

### Elemental distribution and osmotic gradients across the neural retinae

Our elemental microanalysis of light-adapted frozen hydrated retina has shown (see Figure 5) that the intracellular concentrations of Na, K, and Cl, in the inner segments (IS) of the photoreceptors are very similar to that of the bipolar and amacrine cells in the INL though substantially different from the concentrations in the inner proximal layers of the IPL and the large ganglion cell layer.

Indeed, the concentrations of Na, K, and Cl in individual large ganglion cells were unusual (Na 91, K 51, and Cl 45 mmol l^−1^) showing high Na and low K concentrations and a high water content (81 weight percent) as previously described by Marshall and Crewther (2021). Figures 5 and 6 demonstrate that although the content of K, Na, and Cl vary slightly across the five distinguishable horizontal layers in the frozen hydrated IPL (Dreher et al., 1994), the water content (O content; see Figure 6) and higher osmotic concentrations of [K + Na] parallel each other from the ganglion cell layer to the outer nuclear layer but diverge significantly across the photoreceptor outer segmental and apical RPE regions (see Figure 7) and at the vitreal/nerve fiber layer around the aquaporin AQP4 receptors on Müeller cells endfeet (Pannicke et al., 2004; Goodyear et al., 2008). The gradients of the osmotic and water concentration curves are what might be expected for intracellular measures of elements in cells likely to be adjacent to Müeller cells that are known to conduct K^+^ bidirectionally and to be physiologically involved in controlling the osmotic and ionic homeostasis of the extracellular retina (Newman, 1987; Newman and Reichenbach, 1996; Dmitriev et al., 1999; Nagelhus et al., 1999; Bringmann et al., 2006; Goodyear et al., 2008; Netti et al., 2018; Reichhart and Strauß, 2020) from the vitreal border to the hypo-osmotic (relative to plasma) environment of the outer retinal complex of photoreceptor/subretinal space and RPE cells following shifts in light modulated activity (Dmitriev et al., 1999). To attenuate the dynamic osmotic changes initiated by light dark transitions the outer regions of the Müeller cells are reported to swell and release taurine and glutamate along with activation of K and Cl channels to enable regulatory volume control (Netti et al., 2018). Interestingly comparison of water and chloride concentration across the retina (see Figure 7B) indicates that intracellular chloride ion parallels water content across the inner retina to the apical region of the RPE where chlorine concentration increases and water concentration decreases whereas these concentrations are reversed at the RPE basal region. This suggests that the outer retinal water content is more dependent on active shunting of Cl^−^ than in inner retinal neurons or intracellularly across the extent of the Müeller cells.

**Figure 9:**
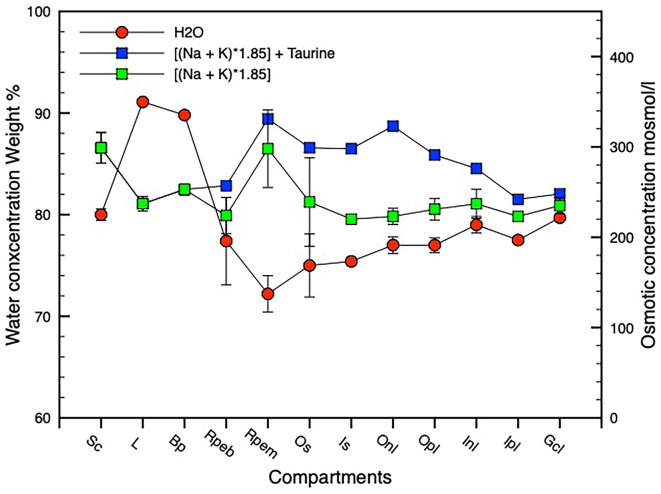
Graph showing water and osmotic concentration gradients across the retinal complex. *n* = 3, SD error bars. An osmotic gradient is shown assuming that taurine acts as an osmolyte using taurine concentration values derived from taurine data in Orr et al. (1976). *n* = 3, SD error bars. Compartment abbreviations on X-axis as in Figure 3A.

Elemental and osmotic analysis of the outer retina/RPE complex demonstrated a sharp decrease in [K] (96–76 mmol l^−1^) and S (89–59 mmol l^−1^) and a relative increase in Na (33–53 mmol l^−1^) and Cl (38–44 mmol l^−1^) between the inner and outer segments of the photoreceptors with a high concentration of K (101 mmol l^−1)^ and slightly higher concentrations of Na 60, and Cl 54 mmol l^−1^ in the melanosomes of the apical region of the RPE cells and lower concentrations of Na 53, K 69, and Cl 48 mmol l^−1^ near the basal membranes. The corresponding changes in osmotic concentrations from slightly hypo-osmotic to blood plasma across the inner retina and an increase to hyper-osmotic in melanosome regions in the apical regions and a return to hypo-osmotic towards the basal membrane support expectations of fluid flow away from the higher concentration of apical K (Miller and Steinberg, 1977; Gallemore et al., 1997; Palmiere and Mangin, 2015) across the RPE and into the choroid. Interestingly, the concentration of S that is often associated with taurine content mimics that of the osmotic concentrations and is opposite to that of water concentration (see Figures 6 and 9).

As shown in Table 2, our results in chick are similar to the single ion electrode dynamic intracellular concentrations estimated previously by Miller and Steinberg (1977) and Mcbrien and Gentle (2001) in isolated frog RPE cells though much higher than that measured in isolated Bullfrog RPE cells (15–20 mmol l^−1^; Wiederholt and Zadunaisky, 1984; La Cour, 1992). The direction of fluid flow is also supported by the greater weight percent of water in the more posterior regions of the choroid, Bruch’s membrane, and the basal area of the RPE compared to apical RPE areas. Chlorine content was close to that measured by Adorante and Miller (1990) in live isolated bovine RPE cells (60 mmol l^−1^). Previous work on other types of living dynamic preparations, e.g., rat liver and heart muscle (Von Zglinicki and Bimmler, 1987), suggests that electroneutrality in RPE cells could, in part, be provided by negative charges on phosphate groups since P concentration was high in these compartments.

### Melanosomes of the microvilli of RPE as potential ion reservoirs in daylight

Both the freeze-substituted and bulkfrozen preparations confirm that the microvilli regions of the apical RPE contain high concentrations of C, Na, K, Cl, and Ca and Zn and low concentrations of S and Mg in daylight conditions. Previous microanalytical investigations of melanosomes in the RPE of various vertebrates (Panessa and Zadunaisky, 1981; Ulshafer et al., 1990; Mishima et al., 1999; Salceda and Sanchez-Chavez, 2000; Eibl et al., 2006; Biesemeier et al., 2011a, 2012) have also reported the presence of S, Ca, Cu, Zn, and Fe though not the important diffusible elements Na and K (White, 1958; Yamada and Ishikawa, 1977; Samuelson et al., 1993; Biesemeier et al., 2018) in aqueous preparations potentially leading to significant variation in measurements. Interestingly early work by Panessa and Zadunaisky (1981) and Salceda and Sanchez-Chavez (2000) has suggested that RPE melanosomes serve as a reservoir for cytoplasmic Ca while this study suggests that K and Na are also likely to be stored in the light-adapted melanosomes and be transportable into the subretinal space during light-dark transitions in daytime, and possibly associated with taurine that is known to be related to K^+^ and circadian rhythms in the pineal body of the rat (Grosso et al., 1978). The affinity of melanin for metal ions is probably due to free negatively charged carboxyl groups on melanin (Larsson and Tjalve, 1978; Hong and Simon, 2007) which would further suggest that the K gradient in the RPE may be due to a Donnan-like association of K^+^ with fixed negative charges in the melanosomes (Hillenkamp et al., 2004a, b).

### Transretinal fluid movements if taurine included

Cell volume regulation throughout the body has long been associated with taurine (Guizouarn et al., 2000; see review Pasantes-Morales and Schousboe, 1997), which is a common amino acid in the retina and where it has been localized to retinal photoreceptor IS, bipolar cells of the ONL, ganglion cells, Müeller cells (Orr et al., 1976; Lake and Verdone-Smith, 1989; Ripps and Shen, 2012) and the RPE (El-Sherbeny et al., 2004). This localization of taurine in the retina also mimics the elemental distribution of S as we have documented in the ONL and OPL and in the RPE. Thus to estimate how the inclusion of taurine might affect the overall osmotic profile shown in Figure 7A we have converted the taurine concentrations reported by Orr et al. (1976) to mosmol l^−1^ using the H_2_O concentrations measured here and added to the osmotic concentrations measured from Na and K as illustrated in Figure 9. What is immediately apparent is that the inclusion of taurine as an osmolyte leads to a more gradual increase in osmotic concentration from the ganglion cell layer to the outer segments of the photoreceptor/apical RPE region while the H_2_O concentration decreases gradually from the ganglion cell layer to the RPE apical region and increases rapidly across the RPE from apical to basal regions, suggesting that water in the normal chick eye may be osmotically transported from vitreous to the outer segments of the photoreceptors from whence transretinal water is likely to cross the apical membrane of the RPE cells in response to the Na and K derived osmotic gradient.

**Table 2:** Comparison of intracellular ion concentrations.

**Authors**	**Cell source**	**Na**	**Cl**	**K**
Present investigation x-ray	RPE apical Chick	60	54	101
Present investigation x-ray	RPE basal Chick	53	48	69
Miller and Steinberg (1977) ise	RPE isolated Frog			110
Wiederholt and Zadunaisky (1984); La Cour (1992) ise	RPE isolated Bullfrog			15–20
Adorante and Miller (1990) ise	RPE isolated Bullfrog	14	27	110
Joseph and Miller (1991) ise	RPE isolated Bovine		60	
Present investigation x-ray	IS Chick	23	28	96
Somlyo and Walz, 1985 x-ray	IS isolated Frog	16	37	145
Somlyo and Walz, 1985 x-ray	OS isolated Frog	22	34	131
Present investigation x-ray	INL bipolar cells Chick	26	24	102
Fujimoto et al. (1992) ise	INL horizontal cells Stingray		130	
Djamgoz and Laming (1987a,b) ise	INL horizontal cells Cyprinid fishes			57/54
Present investigation x-ray	GClarge Chick	91	45	51

Concentrations in mmol l^−1^, ise, ions elective electrode; x-ray, x-ray microanalysis; RPE, retina pigmented epithelium; IS, inner segment of photoreceptor; OS, outer segment of photoreceptor; INL, inner nuclear layer; GC, large ganglion cell.

### Mechanisms of outer retinal fluid movement

Our findings support Hamann, who has previously argued that osmosis alone cannot account for the movement of water from the retinal compartment to the choroid because the retinal compartment is hyperosmotic to the choroid, largely due to lactate in the RPE (Hamann, 2002). Here we see that in the chick, the apical RPE is hyperosmotic to the choroid even without taking lactate into account and that the basal RPE is iso- or slightly hyperosmotic if taurine behaves as an osmolyte. If lactate (Adler and Southwick, 1992) is taken into account, the osmotic gradient between the RPE and choroid would be even steeper, suggesting that water could possibly enter the RPE by osmosis but not exit it by the same simple process. Thus exit of water from the RPE will depend on energy requiring transport systems as suggested by Stern et al. (1980) and Hamann (2002) and consistent with La Cour (1992) who found that the osmotic water permeability of the apical membrane of the RPE cells is greater than that of the basal membrane.

Lastly, our analysis suggests that the cartilaginous layer of the sclera is possibly hyperosmotic to the choroid, due to the high scleral concentrations of S and Na found here and in typical cartilage that consists of abundant sulfated glycosaminoglycan. These highly negatively charged glycosaminoglycan chains are known to interact with Na in the interstitial fluid to form a Donnan osmotic pressure that resists compressive forces on the cartilage (Pando et al., 2017) and is hyperosmotic to the choroid. In chickens the cartilage has been shown to have an increased glycosaminoglycan content in response to induced myopia (Rada et al., 1991; Nickla et al., 1997), suggesting that the cartilage may absorb more water by osmosis. Such a fluid uptake may be related to the reported shrinkage of the choroid (Liang et al., 1995, 2004) in induced myopic eyes in the chick.

#### Strength and limitations

Elemental micro analysis (EDX) in frozen hydrated tissue is both the major strength and the major limitation of this study. The use of light-adapted frozen hydrated tissue has allowed us to identify, qualify and quantify the relative elemental and hydration concentrations across the entire posterior eye cup and neurochemically support many of the current physiologically postulated mechanisms. In particular, the use of EDX on the scanning electron microscope has facilitated the identification of K and Ca in the melanin granules of the melanosomes and confirmed the melanosomes as a storage reservoir for important osmoregulatory ions Na and K at least in daylight conditions. However, EDX of frozen hydrated tissue lacks the sensitivity of freeze-dried preparations to identify trace elements such as Zn.

The major limitation of EDX is that it is an anatomical technique that allows measurement of static osmotic gradients and tissue hydration at a particular point in time but it cannot monitor dynamic physiological adaptation to environmental stimulus. However, we did illustrate in Tables s 1 and 2 the similarity of our findings to those found physiologically in several species when using ion-selective electrodes in single cells and believe this allows us to comment on the feasibility of physiological hypotheses relating to mechanisms.

#### Implications for medical science

Although comparatively little has been known about how ocular growth, optical clarity, and transretinal fluid efflux are established, and how such mechanisms could contribute to daily circadian changes in axial length and refractive power of eyes, our determination of individual ionic concentrations, osmotic and water gradients across the light-adapted chick eye, offers substantial morphological support for early physiological models of retinal fluid (see reviews Gallemore et al., 1997; Crewther, 2000). Furthermore, our new data concur with more recent molecular considerations (see review Reichhart and Strauß, 2020) and is particularly important for a new understanding of genomic and proteomic data pertaining to the development of myopia (shortsightedness) and associated correlations with axial length (Vocale et al., 2021). Indeed our data regarding Cl^−^ and water gradients in the outer retina and RPE enhance and suggest cellular localization of the findings of Vocale et al. (2021). This is clinically important given that abnormally large eyes are the hallmark of clinical myopia that is now accepted as affecting 1.5 billion people while increasing in prevalence and as the greatest risk factor for severe ophthalmic disorders including blindness (Holden et al., 2016).

## Conclusions

EDX of light-adapted frozen hydrated chick eyes has allowed localization, identification, and quantification of the discrete distribution pattern of most important biological elements across the 16 layers of the posterior eye and the relationship of these measures to earlier ion selective electrode research. Osmotic gradients based on Na^+^ and K^+^, and tissue hydration based on O concentration have been calculated and shown to support a hypothesis of gradually increasing osmotic gradient and associated hydration across the inner retina to the photoreceptor/apical RPE region where the rapid increases in concentrations of K^+^ and H^+^ and Cl^−^ are associated with electrogenic Na/K/ATPase and many other ion transporting mechanisms. The high Na and K concentrations in the apical region of the RPE appear to be at least partially derived from the melanosomes that appear in daylight to act as storage reservoirs for these metal ions. Such close colocalizations suggest that transretinal water is likely to cross the apical membrane from the retina into the RPE cells down the Na^+^ and K^+^ derived osmotic concentration gradient and leave the RPE for the choroid across the basal membrane down the extracellular Cl^−^ derived osmotic concentration gradient that has been shown physiologically to be sustained by the ion channels of the basal membrane.

## Data Availability Statement

The original contributions presented in the study are included in the article, further inquiries can be directed to the corresponding author.

## Ethics Statement

The animal study was reviewed and approved by La Trobe University Animal Ethics Committee.

## Author Contributions

Both authors have been involved with the conception, planning, interpretation, and writing. AM was responsible for electron microscopy and elemental microanalysis. All authors contributed to the article and approved the submitted version.

## Funding

No external funds have been available for this research conceived and completed by two Emeritus Professors.

## References

[B1] Adler A. J. Southwick R. E. (1992). Distribution of glucose and lactate in the interphotoreceptor matrix. Ophthalmic. Res. 24, 243–252. 10.1159/000267174 1436983

[B2] Adorante J. S. Miller S. S. (1990). Potassium-dependent volume regulation in retinal pigment epithelium is mediated by Na,K,Cl cotransport. J. Gen. Physiol. 96, 1153–1176. 10.1085/jgp.96.6.1153 2286831 PMC2229036

[B3] Biesemeier A. Eibl O. Eswara S. Audinot J. N. Wirtz T. Schraermeyer U. (2018). Transition metals and trace elements in the retinal pigment epithelium and choroid: correlative ultrastructural and chemical analysis by analytical electron microscopy and nano-secondary ion mass spectrometry. Metallomics 10, 296–308. 10.1039/c7mt00259a 29327028

[B4] Biesemeier A. Julien S. Kokkinou D. Schraermeyer U. Eibl O. (2012). A low zinc diet leads to loss of Zn in melanosomes of the RPE but not in melanosomes of the choroidal melanocytes. Metallomics 4, 323–332. 10.1039/c2mt00187j 22327165

[B5] Biesemeier A. Schraermeyer U. Eibl O. (2011a). Chemical composition of melanosomes, lipofuscin and melanolipofuscin granules of human RPE tissues. Exp. Eye Res. 93, 29–39. 10.1016/j.exer.2011.04.004 21524648

[B6] Biesemeier A. Schraermeyer U. Eibl O. (2011b). Quantitative chemical analysis of ocular melanosomes in stained and non-stained tissues. Micron 42, 461–470. 10.1016/j.micron.2011.01.004 21330141

[B7] Bringmann A. Pannicke T. Grosche J. Francke M. Wiedemann P. Skatchkov S. N.. (2006). Muller cells in the healthy and diseased retina. Prog. Retin Eye Res. 25, 397–424. 10.1016/j.preteyeres.2006.05.003 16839797

[B8] Button K. S. Ioannidis J. P. Mokrysz C. Nosek B. A. Flint J. Robinson E. S.. (2013). Power failure: why small sample size undermines the reliability of neuroscience. Nat. Rev. Neurosci. 14, 365–376. 10.1038/nrn3475 23571845

[B9] Cajal S. L. C. (1892). La retine des vertebres. La Cellule, 9, 17–257.

[B10] Cesetti T. Ciccolini F. Li Y. (2011). GABA not only a neurotransmitter: osmotic regulation by GABA_A_R signaling. Front. Cell Neurosci. 6:3. 10.3389/fncel.2012.00003 22319472 PMC3268181

[B9900] Chahine N. O. Chen F. H. Hung C. T. Ateshian G. A. (2005). Direct measurement of osmotic pressure of glycosaminoglycan solutions by membrane osmometry at room temperature. Biophys. J. 89, 1543–1550. 10.1529/biophysj.104.057315 15980166 PMC1366659

[B11] Chakraborty R. Ostrin L. A. Nickla D. L. Iuvone P. M. Pardue M. T. Stone R. A. (2018). Circadian rhythms, refractive development and myopia. Ophthalmic Physiol. Opt. 38, 217–245. 10.1111/opo.12453 29691928 PMC6038122

[B12] Country M. W. (2017). Retinal metabolism: a comparative look at energetics in the retina. Brain Res 1672, 50–57. 10.1016/j.brainres.2017.07.025 28760441

[B14] Crewther D. P. (2000). The role of photoreceptors in the control of refractive state. Prog. Retin. Eye Res. 19, 421–457. 10.1016/s1350-9462(00)00004-5 10785617

[B15] Crewther S. G. Liang H. Junghans B. M. Crewther D. P. (2006). Ionic control of ocular growth and refractive change. Proc. Natl. Acad. Sci. U S A 103, 15663–15668. 10.1073/pnas.0607241103 17023537 PMC1622878

[B16] De Robertis E. Lasansky A. (1965). Ultrastructure and chemical organization of the photoreceptors. Arch. Oftalmol. B Aires 40, 111–123. 5844977

[B1042] Djamgoz M. B. Laming P. J. (1987a). Intracellular potassium activities of horizontal cells and extracellular potassium activity in isolated retinae of a cyprinid fish. Vis. Res. 27, 711–721. 10.1016/0042-6989(87)90068-x 3660632

[B10420] Djamgoz M. B. Laming P. J. (1987b). Micro-electrode measurements and functional aspects of chloride activity in cyprinid fish retina: extracellular activity and intracellular activities of L- and C-type horizontal cells. Vis. Res. 27, 1481–1489. 10.1016/0042-6989(87)90157-x 3445482

[B17] Dmitriev A. V. Govardovskii V. I. Schwahn H. N. Steinberg R. H. (1999). Light-induced changes of extracellular ions and volume in the isolated chick retina-pigment epithelium preparation. Vis. Neurosci. 16, 1157–1167. 10.1017/s095252389916615x 10614595

[B18] Dowling J. E. (1970). Organization of vertebrate retinas. Invest. Ophthalmol. 9, 655–680. 4915972

[B19] Dreher Z. Distler C. Dreher B. (1994). Vitread proliferation of filamentous processes in avian Muller cells and its putative functional correlates. J. Comp. Neurol. 350, 96–108. 10.1002/cne.903500107 7860802

[B20] Edelman J. L. Miller S. S. (1991). Epinephrine stimulates fluid absorption across bovine retinal pigment epithelium. Invest. Ophthalmol. Vis. Sci. 32, 3033–3040. 1657816

[B21] Eibl O. Schultheiss S. Blitgen-Heinecke P. Schraermeyer U. (2006). Quantitative chemical analysis of ocular melanosomes in the TEM. Micron 37, 262–276. 10.1016/j.micron.2005.08.006 16364648

[B22] El-Sherbeny A. Naggar H. Miyauchi S. Ola M. S. Maddox D. M. Martin P. M.. (2004). Osmoregulation of taurine transporter function and expression in retinal pigment epithelial, ganglion and muller cells. Invest. Ophthalmol. Vis. Sci. 45, 694–701. 10.1167/iovs.03-0503 14744916 PMC3724466

[B99001] Fujimoto M. Yanase H. Katayama J. Toyoda J. (1992). Intracellular and extracellular chloride ions in the horizontal cells of the stingray retina. Jpn. J. Physiol. 42, 525–533. 10.2170/jjphysiol.42.525 1434109

[B23] Gallemore R. P. Hughes B. A. Miller S. (1997). Retinal pigment epithelial transport mechanisms and their contribution to the electroretinogram. Prog. Retin. Eye Res. 16, 509–566.

[B24] Gilles R. (1979). “Intracellular organic osmotic effectors,” in Mechanisms of Osmoregulation in Animals, ed R. Gilles (Chichester: John Wiley & Sons), 111–154.

[B25] Gong H. Kinoshita A. Amemiya T. Kishikawa T. Takaya K. Tozu M.. (2002). Age-related changes of trace elements and vitamins in normal rat retina - time-of-flight secondary ion mass spectrometry study. Invest. Ophthalmol. Vis. Sci. 43:2796.

[B26] Goodyear M. J. Junghans B. M. Giummarra L. Murphy M. J. Crewther D. P. Crewther S. G. (2008). A role for aquaporin-4 during induction of form deprivation myopia in chick. Mol. Vis. 14, 298–307. 18334967 PMC2254964

[B27] Grosso D. S. Bressler R. Benson B. (1978). Circadian rhythm and uptake of taurine by the rat pineal gland. Life Sci. 22, 1789–1798. 10.1016/0024-3205(78)90594-5 672428

[B28] Grubman A. Guennel P. Vessey K. A. Jones M. W. James S. A. De Jonge M. D.. (2016). X-ray fluorescence microscopic measurement of elemental distribution in the mouse retina with age. Metallomics 8, 1110–1121. 10.1039/c6mt00055j 27481440

[B29] Guizouarn H. Motais R. Garcia-Romeu F. Borgese F. (2000). Cell volume regulation: the role of taurine loss in maintaining membrane potential and cell pH. J. Physiol. 523, 147–154. 10.1111/j.1469-7793.2000.t01-1-00147.x 10673551 PMC2269780

[B30] Halsey L. G. Curran-Everett D. Vowler S. L. Drummond G. B. (2015). The fickle P value generates irreproducible results. Nat. Methods 12, 179–185. 10.1038/nmeth.3288 25719825

[B31] Hamann S. (2002). Molecular mechanisms of water transport in the eye. Int. Rev. Cytol. 215, 395–431. 10.1016/s0074-7696(02)15016-9 11952236

[B32] Heinrich K. F. J. (1991). “Strategies for electron probe data reduction,” in Electron Probe Quantitation, ed Newbury K. H. D. (NY and London: Plenum Press), 9–18.

[B33] Hillenkamp J. Hussain A. A. Jackson T. L. Constable P. A. Cunningham J. R. Marshall J. (2004a). Compartmental analysis of taurine transport to the outer retina in the bovine eye. Invest. Ophthalmol. Vis. Sci. 45, 4099–4105. 10.1167/iovs.04-0624 15505061

[B34] Hillenkamp J. Hussain A. A. Jackson T. L. Cunningham J. R. Marshall J. (2004b). Taurine uptake by human retinal pigment epithelium: implications for the transport of small solutes between the choroid and the outer retina. Invest. Ophthalmol. Vis. Sci. 45, 4529–4534. 10.1167/iovs.04-0919 15557464

[B35] Holden B. A. Fricke T. R. Wilson D. A. Jong M. Naidoo K. S. Sankaridurg P.. (2016). Global prevalence of myopia and high myopia and temporal trends from 2000 through 2050. Ophthalmology 123, 1036–1042. 10.1016/j.ophtha.2016.01.006 26875007

[B36] Hong L. Simon J. D. (2007). Current understanding of the binding sites, capacity, affinity and biological significance of metals in melanin. J. Phys. Chem. B 111, 7938–7947. 10.1021/jp071439h 17580858 PMC2533804

[B37] Huxtable R. J. (1992). Physiological actions of taurine. Physiol. Rev. 72, 101–163. 10.1152/physrev.1992.72.1.101 1731369

[B1040] Joseph D. P. Miller S. S. (1991). Apical and basal membrane ion transport mechanisms in bovine retinal pigment epithelium. J. Physiol. 435, 439–463. 1722821 10.1113/jphysiol.1991.sp018518 PMC1181470

[B38] Junghans B. M. Wadley R. B. Crewther S. G. Crewther D. P. (1999). X-ray elemental analysis differentiates blood vessels and lymphatic vessels in the chick choroid. Aust. N Z J. Ophthalmol. 27, 244–246. 10.1046/j.1440-1606.1999.00185.x 10484204

[B39] Kim I. B. Lee E. J. Oh S. J. Park C. B. Pow D. V. Chun M. H. (2002). Light and electron microscopic analysis of aquaporin 1-like-immunoreactive amacrine cells in the rat retina. J. Comp. Neurol. 452, 178–191. 10.1002/cne.10359 12271491

[B40] La Cour M. (1992). Cl- transport in frog retinal pigment epithelium. Exp. Eye Res. 54, 921–931. 10.1016/0014-4835(92)90156-m 1381683

[B41] Lake N. Verdone-Smith C. (1989). Immunocytochemical localization of taurine in the mammalian retina. Curr. Eye Res. 8, 163–173. 10.3109/02713688908995188 2714101

[B42] Larsson B. Tjalve H. (1978). Studies on the melanin-affinity of metal ions. Acta Physiol. Scand. 104, 479–484. 10.1111/j.1748-1716.1978.tb06303.x 726939

[B43] Lasansky A. (1965). Functional implications of structural findings in retinal glial cells. Prog. Brain Res. 15, 48–72. 10.1016/s0079-6123(08)60939-5 5856494

[B44] Lauber J. K. Shutze J. V. (1964). Accelerated growth of embryo chicks under the influence of light. Growth 28, 179–190. 14210735

[B45] Liang H. Crewther D. P. Crewther S. G. Barila A. M. (1995). A role for photoreceptor outer segments in the induction of deprivation myopia. Vis. Res. 35, 1217–1225. 10.1016/0042-6989(94)00241-d 7610583

[B46] Liang H. Crewther S. G. Crewther D. P. Junghans B. M. (2004). Structural and elemental evidence for edema in the retina, retinal pigment epithelium and choroid during recovery from experimentally induced myopia. Invest. Ophthalmol. Vis. Sci. 45, 2463–2474. 10.1167/iovs.03-1009 15277465

[B47] Liu J. H. (1998). Circadian rhythm of intraocular pressure. J. Glaucoma 7, 141–147. 10.1097/00061198-199804000-00014 9559503

[B48] Liu J. H. Kripke D. F. Hoffman R. E. Twa M. D. Loving R. T. Rex K. M.. (1998). Nocturnal elevation of intraocular pressure in young adults. Invest. Ophthalmol. Vis. Sci. 39, 2707–2712. 9856781

[B49] Marmor M. F. (1988). New hypotheses on the pathogenesis and treatment of serous retinal detachment. Graefes Arch. Clin. Exp. Ophthalmol. 226, 548–552. 10.1007/BF02169203 3209082

[B50] Marmor M. F. (1990). Control of subretinal fluid: experimental and clinical studies. Eye (Lond) 4, 340–344. 10.1038/eye.1990.46 2199242

[B51] Marmor M. F. (1997). On the cause of serous detachments and acute central serous chorioretinopathy. Br. J. Ophthalmol. 81, 812–813. 10.1136/bjo.81.10.812 9486016 PMC1722036

[B52] Marshall A. T. (1975). “Electron probe x-ray microanalysis,” in Principles and Techniques of Scanning Electron Microscopy, ed Hayat M. A. (NY: Van Nostrand Reinhold), 103–165.

[B53] Marshall A. T. (1980). Freeze-substitution as a preparative technique for biological X-ray microanalysis. Scan Electron Microsc. 1980, 395–408. 7423124

[B54] Marshall A. T. (2017). Quantitative x-ray microanalysis of model biological samples in the SEM using remote standards and the XPP analytical model. J. Microsc. 266, 231–238. 10.1111/jmi.12531 28181671

[B55] Marshall A. T. Crewther S. G. (2021). An x-ray microanalytical method for measuring in vivo element and water concentrations, relating to osmoregulation, in cells and tissues of the posterior eye. J. Microsc. 283, 21–28. 10.1111/jmi.13004 33605443

[B56] Marshall A. T. Goodyear M. J. Crewther S. G. (2012). Sequential quantitative X-ray elemental imaging of frozen-hydrated and freeze-dried biological bulk samples in the SEM. J. Microsc. 245, 17–25. 10.1111/j.1365-2818.2011.03539.x 21981613

[B57] Mcbrien N. A. Gentle A. (2001). The role of visual information in the control of scleral matrix biology in myopia. Curr. Eye Res. 23, 313–319. 10.1076/ceyr.23.5.313.5440 11910519

[B58] Miller S. S. Steinberg R. H. (1977). Passive ionic properties of frog retinal pigment epithelium. J. Membr. Biol. 36, 337–372. 10.1007/BF01868158 302862

[B59] Mishima K. Amemiya T. Takano K. (1999). X-ray microanalysis of melanin granules of retinal pigment epithelium and choroid in hereditary copper deficient mice (macular mice). Exp. Eye Res. 68, 59–65. 10.1006/exer.1998.0592 9986742

[B60] Nagelhus E. A. Amiry-Moghaddam M. Lehmann A. Ottersen O. P. (1994). Taurine as an organic osmolyte in the intact brain: immunocytochemical and biochemical studies. Adv. Exp. Med. Biol. 359, 325–334. 10.1007/978-1-4899-1471-2_33 7887272

[B61] Nagelhus E. A. Horio Y. Inanobe A. Fujita A. Haug F. M. Nielsen S.. (1999). Immunogold evidence suggests that coupling of K^+^ siphoning and water transport in rat retinal Muller cells is mediated by a coenrichment of Kir4.1 and AQP4 in specific membrane domains. Glia 26, 47–54. 10.1002/(sici)1098-1136(199903)26:1<47::aid-glia5>3.0.co;2-5 10088671

[B62] Netti V. Fernandez J. Kalstein M. Pizzoni A. Di Giusto G. Rivarola V.. (2017). TRPV4 contributes to resting membrane potential in retinal muller cells: implications in cell volume regulation. J. Cell Biochem. 118, 2302–2313. 10.1002/jcb.25884 28098409

[B63] Netti V. Pizzoni A. Perez-Dominguez M. Ford P. Pasantes-Morales H. Ramos- Mandujano G.. (2018). Release of taurine and glutamate contributes to cell volume regulation in human retinal Muller cells: differences in modulation by calcium. J. Neurophysiol. 120, 973–984. 10.1152/jn.00725.2017 29790838

[B64] Newman E. (1987). Distribution of potassium conductance in mammalian Muller (glial) cells: a comparative study. J. Neurosci. 7, 2423–2432. 2441009 PMC6568979

[B65] Newman E. Reichenbach A. (1996). The Muller cell: a functional element of the retina. Trends Neurosci. 19, 307–312. 10.1016/0166-2236(96)10040-0 8843598

[B66] Nickla D. L. Wildsoet C. Wallman J. (1997). Compensation for spectacle lenses involves changes in proteoglycan synthesis in both the sclera and choroid. Curr. Eye Res. 16, 320–326. 10.1076/ceyr.16.4.320.10697 9134320

[B67] Nuzzo R. (2014). Scientific method: statistical errors. Nature 506, 150–152. 10.1038/506150a 24522584

[B68] Orr H. T. Cohen A. I. Lowry O. H. (1976). The distribution of taurine in the vertebrate retina. J. Neurochem. 26, 609–611. 10.1111/j.1471-4159.1976.tb01519.x 816997

[B69] Palmiere C. Mangin P. (2015). Urea nitrogen, creatinine and uric acid levels in postmortem serum, vitreous humor and pericardial fluid. Int. J. Legal Med. 129, 301–305. 10.1007/s00414-014-1076-z 25194712

[B70] Palsgard E. Lindh U. Juntti-Berggren L. Berggren P. O. Roomans G. M. Grime G. W. (1994a). Proton-induced and electron-induced X-ray microanalysis of insulin-secreting cells. Scanning Microsc. Suppl. 8, 325–332. 7638496

[B71] Palsgard E. Lindh U. Roomans G. M. (1994b). Comparative study of freeze-substitution techniques for X-ray microanalysis of biological tissue. Microsc. Res. Tech. 28, 254–258. 10.1002/jemt.1070280309 8068987

[B72] Pando A. Rigoldi F. Vesentini S. (2017). Osmotic pressure characterization of glycosaminoglycans using full-atomistic molecular models. PeerJ [Preprint]. 10.7287/peerj.preprints.3063v18068987

[B73] Panessa B. J. Zadunaisky J. A. (1981). Pigment granules: a calcium reservoir in the vertebrate eye. Exp. Eye Res. 32, 593–694. 10.1016/s0014-4835(81)80008-5 6972313

[B74] Pannicke T. Iandiev I. Uckermann O. Biedermann B. Kutzera F. Wiedemann P.. (2004). A potassium channel-linked mechanism of glial cell swelling in the postischemic retina. Mol. Cell Neurosci. 26, 493–502. 10.1016/j.mcn.2004.04.005 15276152

[B75] Pasantes-Morales H. Schousboe A. (1997). Role of taurine in osmoregulation in brain cells: mechanisms and functional implications. Amino Acids 12, 281–292.

[B76] Rada J. A. Thoft R. A. Hassell J. R. (1991). Increased aggrecan (cartilage proteoglycan) production in the sclera of myopic chicks. Dev. Biol. 147, 303–312. 10.1016/0012-1606(91)90288-e 1916012

[B77] Reichhart N. Strauß O. (2020). “Regulation of ion transport through retinal pigment epithelium: impact in retinal degeneration,” in Ion Transport Across Epithelial Tissues and Disease. Physiology in Health and Disease, eds Hamilton K. L. Devor D. C. (Cham: Springer). 10.1007/978-3-030-55310-4_9

[B78] Ripps H. Shen W. (2012). Review: taurine: a “very essential” amino acid. Mol. Vis. 18, 2673–2686. 23170060 PMC3501277

[B79] Salceda R. Sanchez-Chavez G. (2000). Calcium uptake, release and ryanodine binding in melanosomes from retinal pigment epithelium. Cell Calcium 27, 223–229. 10.1054/ceca.2000.0111 10858668

[B80] Samuelson D. A. Smith P. Ulshafer R. J. Hendricks D. G. Whitley R. D. H.. (1993). X-ray microanalysis of ocular melanin in pigs maintained on normal and low zinc diets. Exp. Eye Res. 56, 63–70. 10.1006/exer.1993.1009 8432335

[B81] Schaffer S. Takahashi K. Azuma J. (2000). Role of osmoregulation in the actions of taurine. Amino Acids 19, 527–546. 10.1007/s007260070004 11140357

[B82] Schmidt-Nielsen B. (1976). Intracellular concentrations of the salt gland of the herring gull Larus argentatus. Am. J. Physiol. 230, 514–521. 10.1152/ajplegacy.1976.230.2.514 1259030

[B83] Seko Y. Shimokawa H. Pang J. Tokoro T. (2000). Disturbance of electrolyte balance in vitreous of chicks with form-deprivation myopia. Jpn. J. Ophthalmol. 44, 15–19. 10.1016/s0021-5155(99)00177-x 10698020

[B84] Sergeant C. Llabador Y. Deve G. Vesvres M. H. Simonoff M. Yefimova M.. (2001). Iron and other elements (Cu, Zn, Ca) contents in retina of rats during development and hereditary retinal degeneration. Nucl. Instrum. Methods Phys. Res. B: Beam Interact. Mater. Atoms 181, 533–538. 10.1016/s0168-583x(01)00484-0

[B85] Sieving P. A. Steinberg R. H. (1985). Contribution from proximal retina to intraretinal pattern ERG: the M-wave. Invest. Ophthalmol. Vis. Sci. 26, 1642–1647. 4055298

[B86] Smith D. W. Lee C. J. Gardiner B. S. (2020). No flow through the vitreous humor: how strong is the evidence. Prog. Retin. Eye Res. 78:100845. 10.1016/j.preteyeres.2020.100845 32035123

[B87] Sobota A. Burovina I. V. Pogorelov A. G. Solus A. A. (1984). Correlation between potassium and phosphorus content and their nonuniform distribution in Acanthamoeba castellanii. Histochemistry 81, 201–204. 10.1007/BF00490118 6490406

[B88] Somlyo A. P. Walz B. (1985). Elemental distribution in Rana pipiens retinal rods: quantitative electron probe analysis. J. Physiol. 358, 183–195. 10.1113/jphysiol.1985.sp015547 3920385 PMC1193338

[B89] Steinberg R. H. (1985). Interactions between the retinal pigment epithelium and the neural retina. Doc. Ophthalmol. 60, 327–346. 10.1007/BF00158922 3905312

[B90] Stern W. Ernest J. Steinberg R. Miller S. (1980). Interrelationships between the retinal pigment epithelium and the neurosensory retina. Aust. J. Ophthalmol. 8, 281–288. 10.1111/j.1442-9071.1980.tb00284.x 7224983

[B91] Stone R. A. Flitcroft D. I. (2004). Ocular shape and myopia. Ann. Acad. Med. Singap. 33, 7–15. 15008555

[B92] Straub O. (2014). Transport mechanisms of the retinal pigment epithelium to maintain of visual function. Heat Mass Transf. 50, 303–315. 10.1007/s00231-013-1267-z

[B93] Strauss O. (2005). The retinal pigment epithelium in visual function. Physiol. Rev. 85, 845–881. 10.1152/physrev.00021.2004 15987797

[B94] Ugarte M. Grime G. W. Lord G. Geraki K. Collingwood J. F. Finnegan M. E.. (2012). Concentration of various trace elements in the rat retina and their distribution in different structures. Metallomics 4, 1245–1254. 10.1039/c2mt20157g 23093062

[B95] Ugarte M. Grime G. W. Osborne N. N. (2014). Distribution of trace elements in the mammalian retina and cornea by use of particle - induced x-ray emission (PIXE): localization of zinc does not correlate with that of metallothioneins. Metallomics 6, 272–278. 10.1039/c3mt00271c 24226809

[B96] Ulshafer R. J. Allen C. B. Rubin M. L. (1990). Distributions of elements in the human retinal pigment epithelium. Arch. Ophthalmol. 108, 113–117. 10.1001/archopht.1990.01070030119041 2297318

[B97] Vaux D. L. (2012). Research methods: know when your numbers are significant. Nature 492, 180–181. 10.1038/492180a 23235861

[B98] Vocale L. G. Crewther S. Riddell N. Hall N. E. Murphy M. Crewther D. (2021). RNA- seq and GSEA identifies suppression of ligand-gated chloride efflux channels as the major gene pathway contributing to form deprivation myopia. Sci. Rep. 11:5280. 10.1038/s41598-021-84338-y 33674625 PMC7935918

[B1000] Von Zglinicki T. Bimmler M. (1987). The intracellular distribution of ions and water in rat liver and heart muscle. J. Microsc. 146, 77–85. 10.1111/j.1365-2818.1987.tb01328.x 3599070

[B99] Wadley R. Junghans B. Dickson M. Liang H. (2002). A quantitative cryo-scanning X- ray microanalysis protocol for the examination of the eye. Scanning 24, 34–38. 10.1002/sca.4950240105 11866343

[B100] Weiss S. Schaeffel F. (1993). Diurnal growth rhythms in the chicken eye: relation to myopia development and retinal dopamine levels. J. Comp. Physiol. A 172, 263–270. 10.1007/BF00216608 8510054

[B101] White L. P. (1958). Melanin: a naturally occurring cation exchange material. Nature 182, 1427–1428. 10.1038/1821427a0 13600352

[B102] Wiederholt M. Zadunaisky J. A. (1984). Decrease in intracellular chloride activity by furosemide in frog retinal epithelium. Curr. Eye Res. 3, 673–675. 10.3109/02713688409003071 6609051

[B103] Wimmers S. Karl M. O. Strauss O. (2007). Ion channels in the RPE. Prog. Retin Eye Res. 26, 263–301. 10.1016/j.preteyeres.2006.12.002 17258931

[B104] Yamada E. Ishikawa H. (1977). Use of frozen thin sections in element analysis. Acta Histochemica et Cytochemica 10, 260–268. 10.1267/ahc.10.260

